# Supplementary material for: Ecological drivers of CRISPR immune systems
Source: mSystems. 2024 Nov 6;9(12):e00568-24. doi: 10.1128/msystems.00568-24 (PMC11651112; doi:10.1128/msystems.00568-24)
Supplement: Legends — Supplemental data file legends. [file msystems.00568-24-s0002.pdf]

Supplementary dataset legends

Supplementary dataset 1 Genera-level abundance and CRISPR incidence in each 16S rRNA sample of the Tara Oceans Project. These data were used to generate Figure 1A,B and Supplemental Figure S5,S7,S8.

Supplementary dataset 2 Summarized version of Supplemental dataset 1 with one row for each genus that lists the average absolute abundance and sample prevalence count (the number of samples that genus is found in) across the 16S rRNA samples in the Tara Oceans Project. The average is only across samples with abundance >0.

Supplementary dataset 3 Genera-level abundance and CRISPR incidence in each saline-water 16S rRNA sample of the Earth Microbiome Project. These data were used to generate Figures 1C,1D and Supplemental Figure S7.

Supplementary dataset 4 Abundance of each MAGs and whether they encode CRISPR or not in each metagenomic sample of the Tara Oceans Project. These data were used to generate Figure 2 and Supplemental Figure S6. Column 2 ("TOBG") contains a genome identifier using the format: Tara Oceans Binned Genome (TOBG)—Province Abbreviation—Numeric ID (e.g., TOBG_NAT-221).

Supplementary dataset 5 Genera-level abundance and CRISPR incidence in each 16S rRNA sample of the Human Microbiome Project. These data were used to generate Figure 3A,C and Supplemental Figure S1,S3,S4.

Supplementary dataset 6 Shannon diversity and CRISPR incidence of each 16S rRNA sample from the human oral study #1774 in the Earth Microbiome Project. These data were used to generate Figure 3B.

Supplementary dataset 7 Shannon diversity and CRISPR incidence (represented by repeat-mapped read counts) of each metagenomic sample in the Human Microbiome Project. These data were used to generate Figure 4A,B and Supplemental Figure S2.
